# Massive Calcified Epithelioid Hemangioendothelioma With Multifocal Involvement: An Imaging Diagnosis Dilemma and a Rare Case Report

**DOI:** 10.3389/fonc.2021.782970

**Published:** 2021-12-17

**Authors:** Feng Che, Cai wei Yang, Xue Hu, Qian Li, Yi Wei, Xi jiao Liu, Bin Song

**Affiliations:** ^1^ West China School of Medicine, West China Hospital, Sichuan University, Chengdu, China; ^2^ Department of Radiology, West China Hospital, Sichuan University, Chengdu, China

**Keywords:** epithelioid hemangioendothelioma, multifocal involvement, calcification, computed tomography, imaging diagnosis

## Abstract

Epithelioid hemangioendothelioma (EHE) is a rare malignant vascular tumor that develops from vascular endothelial or pre-endothelial cells. More than 60% patients have single-organ involvement, and involvement of multiple organs including the liver, lungs, and bones is extremely rare. The typical radiographic features of EHE include multiple small nodules in both lungs, which are usually located near small- and medium-sized blood vessels and the bronchi, and solitary, multiple, or diffuse lesions located at the hepatic periphery, spreading within the branches of the portal and hepatic veins. Radiologic calcification has been rarely reported in the literature. Here, we firstly described a case of a 53-year-old woman with EHE who presented with lungs, liver, bone, and right hilar lymph node involvement, manifesting as massive calcification on computed tomography. This case reminds physicians that EHE may present with unusual imaging manifestations, like massive calcification, and should be considered during the diagnostic process.

## Introduction

Epithelioid hemangioendothelioma (EHE) is a rare vascular neoplasm with a low-to-intermediate risk of malignancy which was first described in 1975 by Dail and Liebow (1). EHE originates from vascular endothelial cells or pre-endothelial cells, with a prevalence of less than one in one million that the current reported literature is limited to case reports. More than 60% patients have single-organ involvement, most commonly, the liver (34%), followed by the bones (21%) and lungs (19%) (2). And the involvement of multiple organs is extremely rare. Pulmonary epithelioid hemangioendothelioma(PEH) typically appear as perivascular nodules 1–2 cm in size throughout the bilateral lung fields, with a lower lobe predominance (3). Hepatic epithelioid hemangioendothelioma(HEH) commonly demonstrates multifocal nodular presentation, with computed tomography (CT) demonstrating clear margins (4). However, radiologic calcification has been rarely reported in the literature. Here, we have reported a very rare case of multifocal EHE presenting as massive calcifications on CT. The imaging features of this case—multiple nodules and masses accompanied by diffuse and massive calcifications in both lungs and liver as well as involvement of the bone and right hilar lymph nodes—are extremely rare. To the best of our knowledge, this is the first report of an EHE presenting as massive calcification radiologically, with multifocal involvement of the lungs, liver, and bones, which is also rare.

## Case Presentation

A 53-year-old woman was referred to our clinic with waist and back pain and numbness of the lower limbs for more than 1 month. The pain was not related to her posture and became more prominent when she moved. She had a medical history of lumbar disc herniation and no history of trauma. On initial evaluation, her vital signs were stable. Apart from the pain of the waist and back, physical examination revealed unremarkable findings. Routine blood tests were obtained. Further, liver function tests revealed normal results. The blood CA199, CA125, CEA, and AFP levels were also within normal limits.

**Figure 1:**
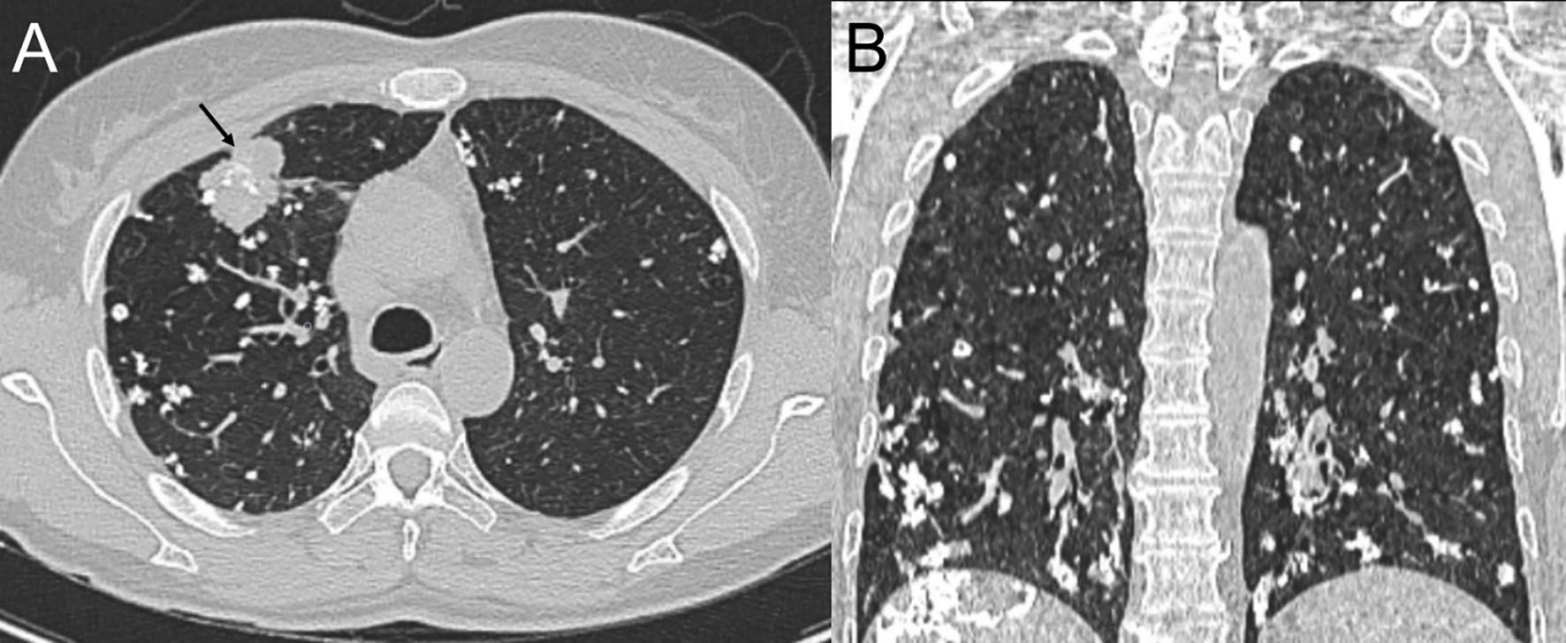
Computed tomography (CT) scan of the chest, lung window, axial view **(A)** and coronal view **(B)**, showing a mass in the right superior lobe (black arrow) and bilateral soft tissue nodules in the lungs with diffuse calcifications adjacent to the bronchioles and small- and medium-sized vessels.

**Figure 2:**
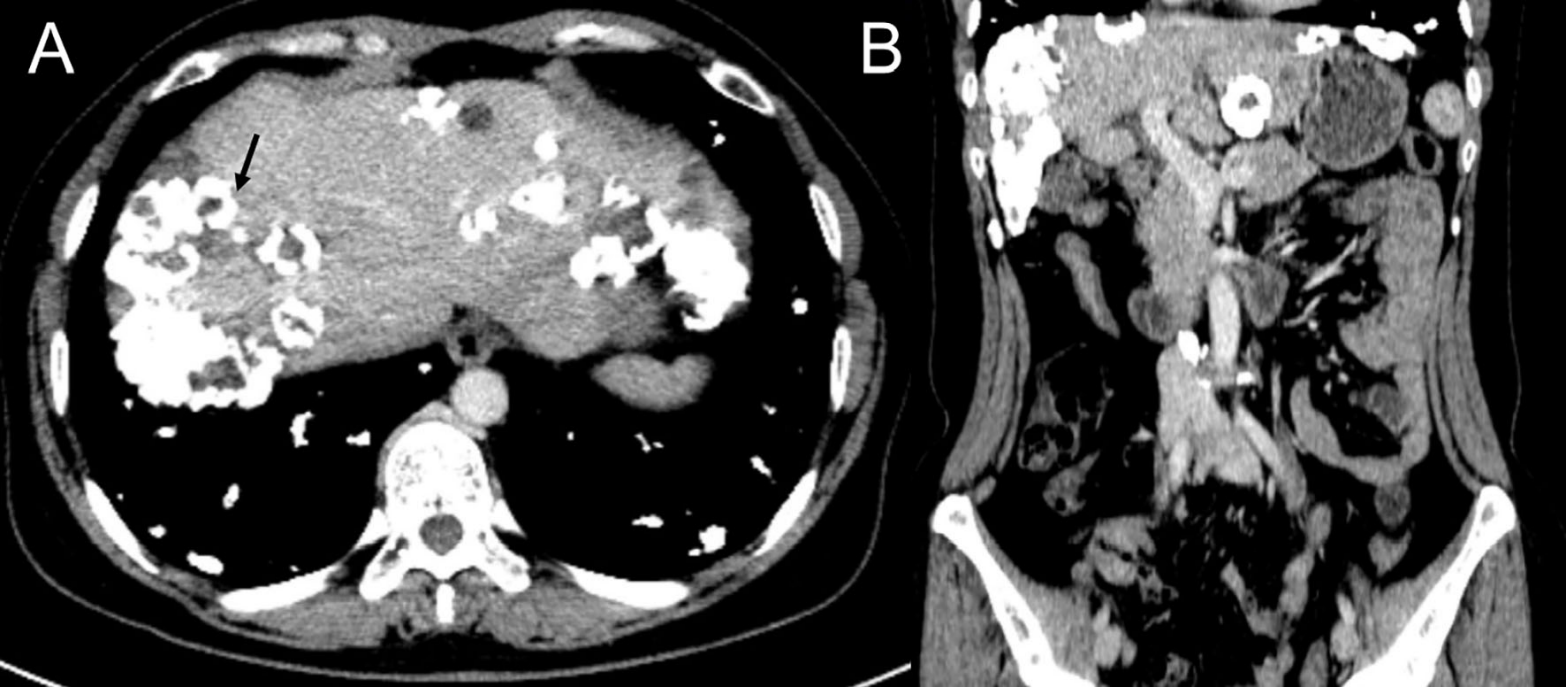
Contrast enhanced CT scan of the abdomen, portal phase, axial view **(A)** and coronal view **(B)**, showing scattered calcified nodules and mass in the liver with the biggest lesion located in the right lobe (black arrow).

**Figure 3:**
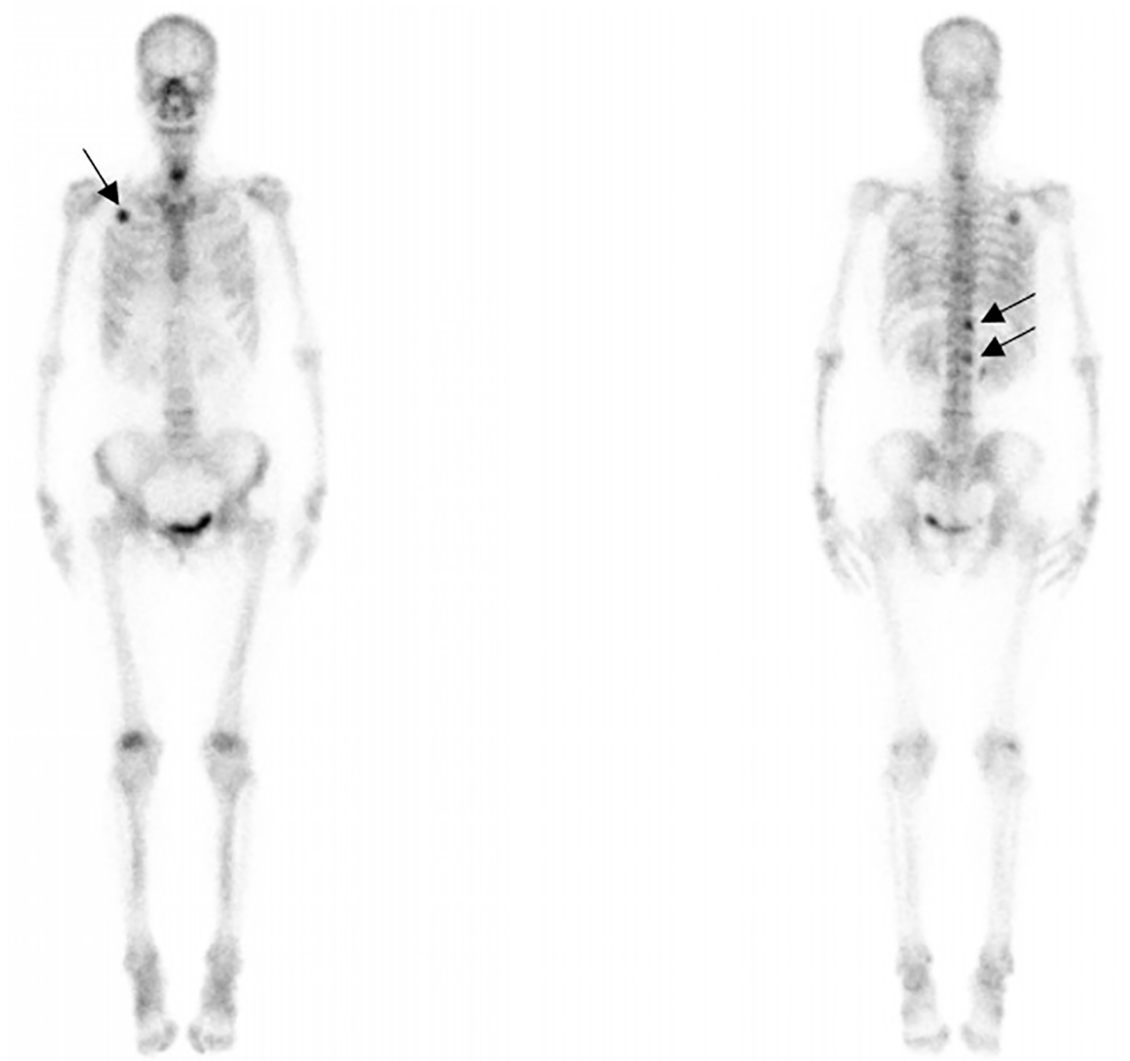
99mTc bone scintigraphy showing intensity at the right second rib, 11th thoracic vertebra, and first lumbar spine (black arrows) accumulation.

**Figure 4:**
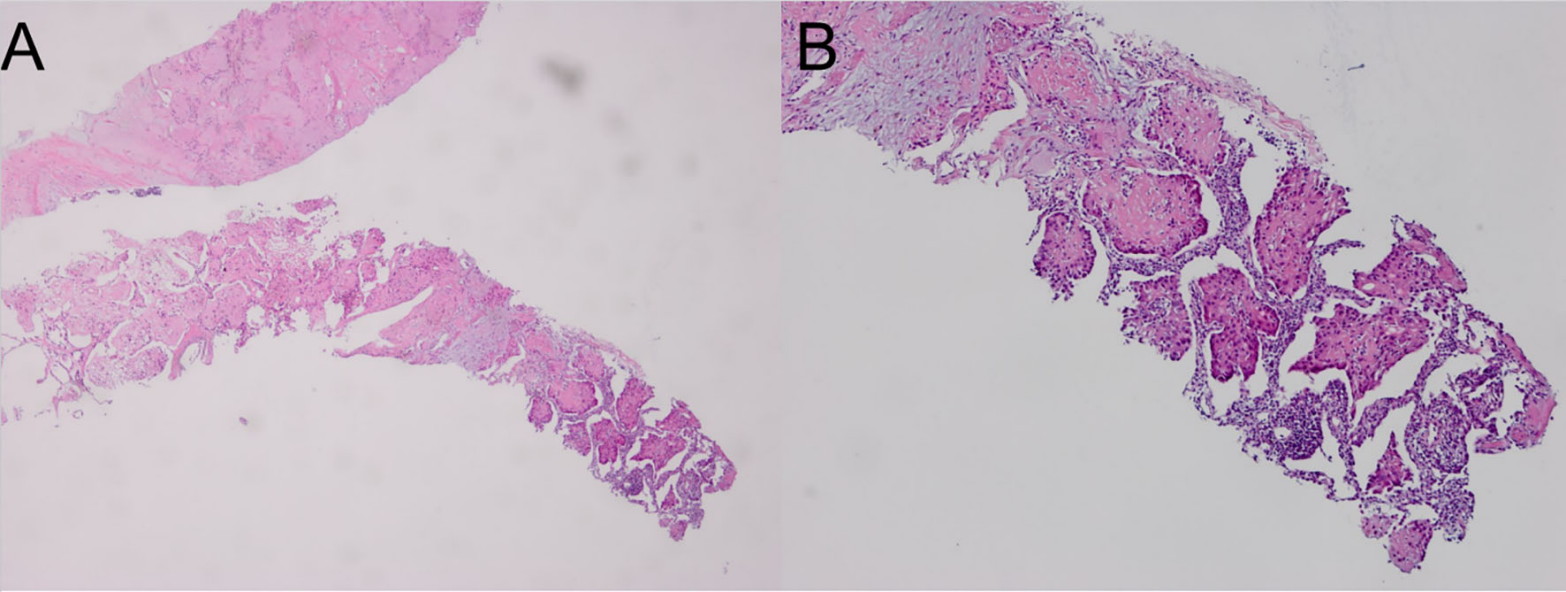
Hematoxylin and eosin staining of the lung biopsy sample showing scattered spindle-shaped tumor cells and epithelioid tumor cells in a myxohyaline stroma. (**A**; hematoxylin and eosin, ×40); higher magnification of the expanded sinusoids lined by large epithelioid tumor cells (**B**; hematoxylin and eosin, ×100).

Computed tomography of the chest revealed scattered pulmonary nodules with calcifications associated with a soft tissue mass measuring 3.3 cm × 2.4 cm and without pleural thickening at the superior lobe of the right lung (**Figure 1**) (SOMATOM definition, Siemens Healthcare, Erlangen, Germany; tube voltage, 100-120 kVp; tube current, 450 mA; slice thickness, 0.625 mm; pitch, 0.992:1; rotation speed: 0.5 s/rot; ASIR-V:30%.). Enlarged lymph nodes of the right hilar were also evident. Abdominal contrast-enhanced CT revealed diffuse lesions with massive calcifications in the liver, which shows faint peripheral enhancement in the arterial phase and low enhancement in the portal phase (Iopromide Injection, Bayer Pharma AG; the arterial phase and portal venous phase were obtained at 25 s and 60 s after contrast injection.). The largest lesion measuring 10.2 cm × 5.9 cm was located in the right lobe of the liver and (**Figure 2**). CT examination also revealed osteolytic lesions with a massive thick sclerotic rim in the right second rib, 11th thoracic vertebra, and first lumbar spine. Bone scintigraphy with 99mTc-methylene diphosphonate showed multiple hypermetabolic activities in the involved bones (**Figure 3**). Cerebral magnetic resonance imaging (MRI) revealed no anomalies. The patient underwent transthoracic needle biopsy of the largest pulmonary lesion located in the right superior lobe. Histopathological analysis revealed epithelioid cells arranged in a glandular pattern with clear cytoplasm (**Figure 4**). Immunohistochemical staining showed that the neoplastic cells were positive for CD31, CD34, CAMTA1, and EMA, but negative for ERG, TFE3, PCK, and desmin, with a Ki-67 index rate of 10%. Histopathological examination indicated a rare low-grade malignant vascular neoplasm, confirming the diagnosis of EHE.

Considering the multiple intra-pulmonary, right hilar lymph node, liver, and bone metastases, the patient was treated with chemotherapy with paclitaxel liposome (240 mg/m^2^; day 1) and carboplatin (550 mg/m^2^; day 1). At 8 months, the patient had completed four cycles of combination therapy. There were no changes in the patient’s disease status on CT at the 8-month follow-up visit.

## Discussion

EHE is a low-grade malignant vascular tumor that is usually located in soft tissues or deep internal structures. The age at diagnosis ranges from 3 years to 86 years, with a peak incidence in middle-aged adults and a mild female predilection (male: female: 1:4) (5, 6). Most patients have nonspecific symptoms on initial presentation with generally indolent clinical activity, but can sometimes demonstrate aggressive clinical behavior (7). Diagnosis of EHE depends on a combination of unique histologic, immunohistochemical, and molecular characteristics since there is nonspecific imaging and the differential diagnosis for EHE is broad. Usually, it is histologically characterized by epithelioid tumor cells that are spread throughout the myxohyaline stroma. The tumor cells are immunohistochemically positive for vascular endothelial markers, including CD31, CD34, cytokeratin, vimentin, and factor VIII antigen. And the Ki-67 index rate of >10%–15% has been shown to be a more aggressive feature (3, 8). Nodules measuring 3 cm were also considered to demonstrate a worse survival (9). Our patient had a pulmonary mass measuring 3.3 cm and a liver mass measuring 10.2 cm as well as bone involvement, further immunohistochemical staining showed Ki-67 index rate of 10%. What is more remarkable is its unique radiographic findings, which appears to be a challenge for clinician to give a consideration of this rare disease.

The typical radiographic findings of PEH are multiple small nodules in both lungs, which are usually located near small- or medium-sized blood vessels and the bronchi. Most PEH lesions are less than 1 cm in diameter, but occasionally, lesions up to 2 cm can occur. This is the first case report of a PEH with rare radiologic features including numerous distinct calcified nodules with largest mass measuring 3.3 cm. This presentation can be easily misdiagnosed as other lung diseases such as disseminated tuberculosis, metastasis (such as metastases secondary to osteosarcoma, mucin-producing carcinoma, thyroid malignancy, and treated metastatic choriocarcinoma), and pulmonary amyloidosis. Radiologic calcifications in PEH are rare, but histological examinations often show nodules with calcified and ossified necrotic centers on microscopy (10). In most cases, patients develop calcifications 10–20 years after diagnosis, the majority of which tend to be punctate calcifications. Our patient was diagnosed with lumbar disc herniation 2 years prior and showed no abnormalities in the lungs at that time. The mechanism of calcification in these nodules is multifactorial which is not precisely understood. Further research is needed to clarify whether this is due to the disease itself or individual heterogeneity.

In EHE, liver is more frequently involved than lung. HEH can be classified as solitary, multiple, or diffuse. The multifocal nodules generally spread within the branches of the portal and hepatic veins and, most commonly, at the periphery. Intratumor calcification, capsular retraction, halo sign, and lollipop sign have been reported as relevant imaging features for distinguishing HEH on CT or MRI. Intratumor calcification is an inconsistent but suggestive feature of HEH and is only seen in 12.7% HEH cases (11, 12). The presence of coalescent lesions is considered a risk factor that is often associated with hepatic and/or portal vein inversion (13–15). Our patient presented with extremely rare imaging features of scattered nodules/masses with extensive calcifications accompanied by coalescence lesions. The tumors mainly located in the periphery without actual bulging of the liver capsule, which is assumed to be a radiologic characteristic of HEH (16). Differential diagnosis of HEH is difficult and should be distinguished from multifocal metastases (such as metastases of breast, adrenal gland, or colon tumors), multiple sclerosing hemangioma, hepatocellular carcinoma (HCC) with hepatic metastases, intrahepatic parasitic or bacterial infections, and others. Typical imaging findings are important for pre-treatment diagnosis, but a combination of clinical examination, medical history, laboratory and pathological assessments are essential for diagnosis. Occasionally, immunohistochemical and molecular biology analysis is needed for that the tumor can be mistaken for HCC or metastatic carcinoma, cholangiocarcinoma, angiosarcoma or undifferentiated sarcomas on histological examination.

Bone EHE accounts for less than 1% of all primary bone neoplasms. Cases occurring in the spine region are especially rare as long tubular bones are more commonly affected (17). The typical radiographic characteristic of bone EHE is an osteolytic lesion with a thin sclerotic rim. Periosteal reactions and matrix calcification are uncommon (3, 18). Our case showed massive high-attenuation lesions in the involved bones with an apparent thick rim that could not be easily differentiated as calcification or ossification. However, high-attenuation lesions are similarly identified with calcification in lung and liver lesions. Multifocal EHE of the bones can be easily misdiagnosed as metastatic tumors, multiple myelomas, or brown tumors owing to the overlapping imaging features, while previous research has indicated that the clustering of multiple lesions in the same anatomic region can help with the diagnosis of a vascular tumor. For bone EHE, whole-body scintigraphy has the advantage of showing the distributional characteristics of multiple lesions. Earlier detection of silent lesions is possible owing to its high sensitivity (19, 20), although the final diagnosis depends on histopathology and immunochemical staining results.

The diagnosis of EHE can be challenging, and treatment options are not standardized. There is limited clinical data to guide treatment choices due to its rarity. Consequently, very few therapeutic options are available and treatment throughout the literature review has been based on individual basis. Surgical resection can achieve good outcomes for patients with a limited number of lesions and no metastases. Chemotherapy or immunotherapy can be considered in patents with asymptomatic metastatic disease (21, 22). Some studies have proven that radiotherapy can help patients with exclusive bone presentations achieve local pain control and better quality of life when combined with bone surgery (23, 24). There are several cases of cancer regression or remission achieved in patients treated with conventional chemotherapy drugs such as carboplatin, etoposide, and paclitaxel (25). Our patient opted for chemotherapy with paclitaxel liposomes and carboplatin, both of which are cell cycle-nonspecific drugs, due to its disseminated and unresectable characteristic. Eight months after treatment, the discomfort caused by lower back distress disappeared. However, there was no change in the patient’s disease status on CT. Treatment evidence for this rare disease is limited to small trials and case series, more clinical trials are needed to identifying new treatment targets.

## Conclusion

In conclusion, this case illustrates a rare manifestation of EHE with multiple organ involvement. Each lesion manifested with an uncommon radiological finding with extensive calcification. Since there are no specific radiological signs, we should be aware of the possible radiological presentations of EHE, which may help with preoperative diagnosis and clinical treatment planning.

## Data Availability Statement

The original contributions presented in the study are included in the article/supplementary material. Further inquiries can be directed to the corresponding author.

## Ethics Statement

The studies involving human participants were reviewed and approved by the West China Hospital of Sichuan University. The patients/participants provided their written informed consent to participate in this study.

## Author Contributions

FC and XH acquired the data. YW, CY, and QL analyzed and interpreted the data. FC and CY conducted the radiological analysis of MRI and CT images, and FC prepared the manuscript. XL and BS revised the manuscript. All authors contributed to the article and approved the submitted version.

## Conflict of Interest

The authors declare that the research was conducted in the absence of any commercial or financial relationships that could be construed as a potential conflict of interest.

## Publisher’s Note

All claims expressed in this article are solely those of the authors and do not necessarily represent those of their affiliated organizations, or those of the publisher, the editors and the reviewers. Any product that may be evaluated in this article, or claim that may be made by its manufacturer, is not guaranteed or endorsed by the publisher.
